# Effect of a mobile app intervention on vegetable consumption in overweight adults: a randomized controlled trial

**DOI:** 10.1186/s12966-017-0563-2

**Published:** 2017-09-15

**Authors:** Sarah Mummah, Thomas N. Robinson, Maya Mathur, Sarah Farzinkhou, Stephen Sutton, Christopher D. Gardner

**Affiliations:** 1 Department of Medicine, Stanford University School of Medicine, Stanford, CA USA; 2 Department of Public Health and Primary Care, University of Cambridge, Cambridge, UK; 3 Department of Pediatrics, Stanford University School of Medicine, Stanford, CA USA

**Keywords:** mHealth, Smartphone, Mobile, Digital, Diet, Nutrition, Vegetables, Behavior change, User-centered design, Design thinking

## Abstract

**Background:**

Mobile applications (apps) have been heralded as transformative tools to deliver behavioral health interventions at scale, but few have been tested in rigorous randomized controlled trials. We tested the effect of a mobile app to increase vegetable consumption among overweight adults attempting weight loss maintenance.

**Methods:**

Overweight adults (n=135) aged 18–50 years with BMI=28–40 kg/m^2^ near Stanford, CA were recruited from an ongoing 12-month weight loss trial (parent trial) and randomly assigned to either the stand-alone, theory-based Vegethon mobile app (enabling goal setting, self-monitoring, and feedback and using “process motivators” including fun, surprise, choice, control, social comparison, and competition) or a wait-listed control condition. The primary outcome was daily vegetables servings, measured by an adapted Harvard food frequency questionnaire (FFQ) 8 weeks post-randomization. Daily vegetable servings from 24-hour dietary recalls, administered by trained, certified, and blinded interviewers 5 weeks post-randomization, was included as a secondary outcome. All analyses were conducted according to principles of intention-to-treat.

**Results:**

Daily vegetable consumption was significantly greater in the intervention versus control condition for both measures (adjusted mean difference: 2.0 servings; 95% CI: 0.1, 3.8, *p*=0.04 for FFQ; and 1.0 servings; 95% CI: 0.2, 1.9; *p*=0.02 for 24-hour recalls). Baseline vegetable consumption was a significant moderator of intervention effects (*p*=0.002) in which effects increased as baseline consumption increased.

**Conclusions:**

These results demonstrate the efficacy of a mobile app to increase vegetable consumption among overweight adults. Theory-based mobile interventions may present a low-cost, scalable, and effective approach to improving dietary behaviors and preventing associated chronic diseases.

**Trial registration:**

ClinicalTrials.gov NCT01826591. Registered 27 March 2013.

## Background

Greater consumption of vegetables and fruits is associated with reduced risks of cardiovascular disease, stroke, cancer, and all-cause mortality [1, 2]. It has been suggested that these protective effects are greater for vegetables than for fruits [1] and follow a dose-response relationship [1–3] with benefits seen in up to 7+ servings daily [1]. However, despite current United States Department of Agriculture (USDA) recommendations to consume 5–6 servings of vegetables per day [4], United States (U.S.) adults consume an average of just 1.7 (SE = 0.03) servings of vegetables (excluding fried potatoes) each day [5]. Behavioral interventions to increase vegetable and fruit consumption have led to modest increases in daily intake [6], but typical face-to-face approaches often cannot be realistically implemented at the population level [6], particularly given the high cost of most current strategies [7] (e.g., time demands, the need for trained staff, etc.) [8].

Mobile applications (apps) present a promising means for delivering scalable behavioral interventions due to their increasing ubiquity, ability to reach individuals at nearly any time or place, and potential to deliver timely feedback, personalization, and interactivity to maximize intervention effectiveness [9]. The availability of health-promoting mobile apps has dramatically increased in recent years [10]. However, most mobile health apps have yet to undergo evaluation in randomized trials or incorporate theory-based strategies known to drive changes in health behaviors [11–13]. Among published mobile app trials, most are feasibility and pilot studies with small sample sizes [14]. Only one published study [15] sought to assess the efficacy of a stand-alone mobile app for increasing vegetable consumption, in which real-time goal setting, self-monitoring, and feedback were tested among low-income racial/ethnic-minority girls; this pilot study showed non-significant small to moderate effects and called for larger-scale testing of similar programs. Thus, the existing evidence has yet to establish whether a mobile app can increase vegetable consumption. More broadly, investigators have called for both theory-informed development and rigorous evaluation of mobile-based interventions [16, 17] using larger sample sizes [14].

A recent pilot study [18] we conducted demonstrated preliminary evidence of the efficacy of Vegethon, a stand-alone, theory-based mobile app, in increasing vegetable consumption among overweight adults [19]. While many public health interventions focus on anticipated long-term health benefits (e.g., reduced risk of cardiovascular disease) to motivate behavior change, Vegethon focuses on making the process of behavior change itself (e.g., increasing vegetable consumption) intrinsically rewarding, through the use of *process motivators* [20] such as fun, surprise, choice, control, and competition [19]. This approach has been introduced as a more effective strategy for designing public health interventions to motivate behavior change [20, 21]. The present study aimed to rigorously assess the efficacy of the Vegethon mobile app among a larger sample of overweight adults attempting weight loss maintenance.

## Methods

### Study design

A randomized controlled study design was used, with a 1:1 allocation ratio of intervention to wait-listed control (8-week delay). The study was conducted at a single research site in Stanford, California.

### Study sample

Participants were overweight adults, recruited in two cohorts from an ongoing 12-month weight-loss trial based at Stanford University (parent trial), in which individuals were 18–50 years of age, had baseline body mass index (BMI) of 28–40 kg/m^2^, were non-diabetic and non-hypertensive, had no cancer or heart, renal, or liver disease, and lived in the geographic area surrounding Stanford. We chose to design and test Vegethon among participants in a weight loss trial because they had already demonstrated motivation to alter their eating behaviors and because the parent trial provided a resource-efficient way of testing an innovative mobile intervention in the setting of a weight loss program. The added eligibility criterion for cohort 1 was ownership of an iPhone and for cohort 2 was ownership of an iPhone or Android phone (as the app became available on Android during the course of the study). This study was implemented during months 5–7 (i.e., the weight loss maintenance phase) of the 12-month parent trial. Parent trial participants were randomized to either a low-fat or a low-carbohydrate diet for 12 months and attended 22 evening classes led by a health educator, in which they were taught how to adhere to their assigned diets. Both parent trial treatment groups were encouraged to eat whole, unprocessed foods and to increase their vegetable intakes in their daily diets. Parent trial participants who chose to concurrently participate in the present study were newly randomized to receive the mobile app or to be placed on a wait-listed control condition. As a result, any potential effects of the parent trial interventions on vegetable consumption were randomly distributed across both intervention and control conditions in the present Vegethon study.

### Randomization and blinding

Randomization was performed by a researcher who had no contact with participants and used a random, computer-generated allocation sequence to assign participants prospectively to each condition. The allocation sequence was designed to ensure balance across mobile intervention assignment groups as well as cohorts, diet group, and health educator within the parent trial. Because the randomized parent trial was underway at the time of the present study, cohort, diet group, and health educator were treated as existing strata, within which participants were assigned to a mobile intervention through a balanced, randomized procedure, using blocks of size 4. This assignment procedure was preferable to simple randomization because it ensured balance in mobile intervention, cohort, diet group, and health educator at any given sample size of this trial. The allocation sequence was stored electronically and concealed from participants, staff, and researchers who had contact with participants. Enrollment and communication with participants was performed by a research assistant who did not play a role in randomization or data analysis. Study staff and researchers who performed data collection (i.e., dietary recall assessors) and analysis were blinded to condition assignment. While participants could not be blinded, they were not told their condition assignment (i.e., “intervention” or “control”) and were instead informed they would be assigned to use the app at parent trial month 5 or 7.

### Procedures

During a pre-randomization orientation session, participants were instructed that the mobile app was intended to support them in increasing their vegetable consumption, and that it was ideally to be used for at least 1–2 min per day. Participants completing a baseline questionnaire were randomized. Those allocated to the intervention condition completed a short online tutorial that described the mobile app and its use. To prevent contamination among conditions, the app was not freely accessible via the App Store or Google Play and was made available to intervention participants through the distribution of single-use registration codes.

The user-centered, theory-based development and final version of Vegethon were shaped by extensive formative research and the Vegethon pilot study [18] and have been described in detail elsewhere [19]. Vegethon was a stand-alone mobile app that enabled goal-setting and self-monitoring of vegetable consumption. Participants set goals for daily quantity (i.e., servings) and variety (i.e., types) of their vegetable consumption and used the central app feature, *Select Veggies*, to tap on any of 30 different vegetable icons (e.g., spinach, beets) to swiftly record the types and servings of vegetables they consumed each day. Non-starchy vegetables with low energy density and high nutrient density were targeted to facilitate weight loss and weight maintenance while maximizing diet quality and nutrient adequacy in the context of the parent trial. Seven ongoing challenges were included that ranged from easy to difficult to perform (e.g., *Breakfast Champ: Eat any vegetable before 11 am*). A leaderboard engaged users in competition against “other Vegethoners” who were “most similar” to them. Just-in-time prompts to log vegetables before going to sleep were delivered most evenings at 9 p.m. through push notifications.

Overall, the app focused on making the *process* of behavior change rewarding [20] (e.g., the fun of receiving a surprise vegetable challenge; the pride in surpassing a peer’s vegetable score). This process motivation strategy stands in contrast to strategies focusing on the eventual outcomes of behavior change (e.g., avoidance of a heart attack) that are often too far in the future to motivate and sustain day-to-day behavior changes [20]. As Robinson has described [20], this process motivation approach is grounded in Bandura’s Social Cognitive Theory [22], which specifies that behaviors are driven by the reciprocal interactions between personal, behavioral, and environmental influences. Vegethon was therefore designed to influence users’ thoughts and beliefs about eating vegetables, provide opportunities for feedback on their behaviors, and create a social environmental that promoted increasing vegetable intake. Vegethon aimed to maximize the four key processes identified by Social Cognitive Theory: attentional, retention (memory), production, and motivational. For example, attention was maximized through prompts, reminders, and a colorful, playful, interactive user interface; retention was promoted through reminders, repetition, memorable graphics and content; production (promoting the transition of thoughts into action) was supported through instructions and tips and by prompting cognitive and behavioral rehearsal with feedback; and motivation for change was maximized through internal and social comparisons, goals, and feedback.

### Measures

The pre-specified primary outcome measure was average daily servings of vegetable consumption, assessed at baseline and 8 weeks post-randomization using an adapted version of the validated semi-quantitative Harvard Food Frequency Questionnaire (FFQ) [23]. The FFQ was selected as the primary outcome due to its: a) specificity to the intervention (i.e., probing each of the vegetables targeted by the intervention vs. holistic probing of all food intake); b) minimal participant burden leading to a reduced amount of missing data; c) high test-retest reliability in the target population; [18] d) evidence of sensitivity to change in the Vegethon pilot study; [18] and e) demonstrated comparable validity to 24-h recalls in measuring vegetable consumption [24]. Commonly used portion sizes for each vegetable (e.g., ½ cup of string beans) were specified, and participants were asked to indicate how often, on average, during the past week, they had consumed each type and amount of food. Eight response categories were possible, ranging from 0 to ≥6 times per day [25]. All 28 questions on vegetable consumption were included from the Harvard FFQ, supplemented by 12 further questions on the consumption of additional vegetables targeted by the intervention. Daily vegetable intake was calculated for each participant following an established method [26] in which the daily frequency of consumption for each vegetable item was multiplied by the number of servings represented by the specified portion size and subsequently combined to yield total daily vegetable servings. Vegetable subgroups were defined *a priori* based on established criteria [27] and included green leafy vegetables, cruciferous vegetables, dark yellow vegetables, tomatoes, other vegetables, and beans. All subgroups excluding beans were combined to yield overall vegetable consumption (primary outcome), and assessed individually as secondary outcomes. Beans, not targeted by the intervention, was included as a comparison measure to detect possible global vegetable reporting bias.

Secondary measures of vegetable consumption were also derived from 24-h dietary recalls at baseline and 5 weeks post-randomization, administered by trained, certified, and blinded interviewers using Nutrition Data System for Research (University of Minnesota Nutrition Coordinating Center) [28, 29]. Three unannounced 24-h recalls were attempted on nonconsecutive days (2 weekdays, 1 weekend) over 2 weeks at each of two time points, for a total of 6 recalls per participant. Average daily servings of four subgroups (dark green vegetables, deep yellow vegetables, tomato, and other vegetables) were combined to yield overall vegetable consumption. Beans was a comparison measure.

App usage was measured using inbuilt software. App usability and satisfaction were assessed using a 21-item questionnaire, administered 8 weeks post randomization to the intervention condition only, and adapted from similar surveys used by King et al. to assess user acceptability of mobile health (mHealth) interventions [30]. Participants were asked to rate their level of agreement or disagreement with each statement on a 5-point Likert-type scale.

### Statistical analysis

*A priori* sample size estimation determined that a sample size of *n* = 128, or 64 per group, would provide at least 80% power to detect a medium effect size of Cohen’s d = 0.5 (one-half standard deviation) [31]. Each analysis used a two-sided alpha = 0.05 threshold for statistical significance. To assess the effect of app assignment on participants’ all-vegetable consumption as measured by FFQ (primary outcome), we fit a linear mixed-effects model to which each subject contributed two observations (at baseline and 8 weeks post-randomization) over the study period. The model contained main effects of app intervention status (an indicator of whether an individual was assigned to the app at the time of observation), time (baseline or 8 weeks post-randomization), sex, parent trial diet group assignment (low-carbohydrate or low-fat), parent trial health educator (4 categories), and parent trial cohort group (2 categories). The main effect of app was the primary effect of interest. To account for correlated observations within subjects, the model also included random intercepts by subject, as well as random slopes over time by subject. We assumed normally distributed error terms. An analysis following intention-to-treat principles was used with multiple imputation for missing data. Five imputed datasets were created using the chained equations method and pooled estimates using Barnard-Rubin adjusted degrees of freedom.

We conducted several pre-specified secondary analyses. We refit the primary analysis model, using alternative secondary outcome measures: [1] all-vegetable consumption as measured by 24-h recalls; [2] each of 5 vegetable subgroups as measured by FFQ; and [3] each of 4 vegetable subgroups as measured by 24-h recalls. We also performed pre-specified analyses of four potential moderators of treatment effects, baseline vegetable consumption, sex, diet group, and health educator, by adding app by possible moderator interaction terms to the model. When a statistically significant interaction was identified, it indicated a possible effect moderator, and the main effect coefficient in the model was re-examined after centering the variables [32]. To investigate the possibility of a dose-response relationship between the amount of app usage and changes in vegetable consumption, we used the Spearman rank correlation coefficient that does not assume normal distributions. Data were collected in 2014–2015 and analyzed in 2015–2016. All primary and secondary analyses followed intention-to-treat principles. Stata (v14.1, StataCorp LP, Texas) and SPSS Statistics software (v22, IBM, New York) were used.

## Results

**Fig. 1:**
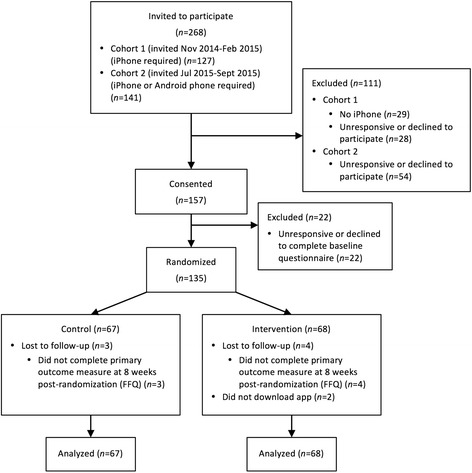
CONSORT flow diagram

Between Nov 3, 2014 and Jan 30, 2016, 135 participants were randomized and analyzed (intervention: 68; control: 67) (Fig. 1). Participant baseline characteristics are presented in Table 1. 100% and 96% of the control condition and 100% and 94% of the intervention condition completed the primary outcome measure (FFQ) at baseline and 8 weeks post-randomization. For the secondary outcome measure (24-h recalls), 96% and 97% of the control condition and 97% and 85% of the intervention condition completed at least one recall at baseline and 5 weeks post-randomization, respectively; and 46% and 40% of the control and intervention conditions, respectively, completed all three recalls at both baseline and 5 weeks post-randomization. Of the 68 participants randomized to the intervention, 43 (63%) downloaded the app within 2 days, 17 (25%) downloaded it after 3–6 days, 6 (9%) downloaded it after 1–5 weeks, and 2 (3%) did not download it. In addition, 51 (75%) used the app to log vegetables at least once, while 17 (25%) did not use the app to log.

**Table 1:** Baseline characteristics, primary and secondary outcome measures for vegetable consumption, and group differences

	Baseline^a^		Post-randomization^a^		Adjusted difference post-randomization^b^	
	Control	App	Control	App	Mean (95% CI)	*p* value
Female, *n* (%)	42 (62.7)	42 (61.8)				
Age, years, M (SD)	40.3 (5.8)	39.4 (6.7)				
Race/ethnicity, *n* (%)						
White	50 (74.6)	41 (60.3)				
Asian	5 (7.5)	5 (7.4)				
Black/African American	5 (7.5)	3 (4.4)				
Other	7 (10.4)	19 (27.9)				
Ethnicity, *n* (%)						
Non-Hispanic	46 (68.7)	51 (75.0)				
Hispanic	21 (31.3)	17 (25.0)				
Education, *n* (%)						
≤ Some college	13 (19.4)	16 (23.5)				
College graduate	27 (40.3)	27 (39.7)				
Post-graduate degree	27 (40.3)	25 (36.8)				
Marital Status, *n* (%)						
Married/partnered	44 (65.7)	37 (54.4)				
Single/never married	16 (23.9)	23 (33.8)				
Separated/divorced	7 (10.4)	8 (11.8)				
Phone, *n* (%)						
iPhone	60 (89.6)	61 (89.7)				
Android	7 (10.4)	7 (10.3)				
FFQ,^c^ *n* (%)	67 (100)	68 (100)	64 (96)	64 (94)		
All vegetables,^d^ M (SD)^g^	8.1 (8.2)	6.7 (5.2)	6.4 (4.3)	7.4 (5.4)	2.0 (0.1, 3.8)^d^	**0.04** ^d^
Green leafy	2.3 (4.2)	1.6 (1.7)	1.5 (1.2)	1.5 (1.3)	0.3 (−0.5, 1.1)	0.42
Cruciferous	0.9 (1.1)	0.9 (1.5)	0.7 (0.7)	1.1 (1.0)	0.5 (0.1, 0.8)	**0.01**
Dark yellow	0.7 (1.5)	0.5 (0.6)	0.5 (0.5)	0.5 (0.6)	0.2 (−0.1, 0.5)	0.23
Tomatoes	0.8 (0.9)	0.6 (0.5)	0.6 (0.4)	0.6 (0.5)	0.1 (−0.1, 0.3)	0.23
Other	3.3 (2.8)	3.2 (2.6)	3.2 (2.5)	3.7 (3.2)	0.8 (−0.1, 1.7)	0.06
Beans (not targeted), M (SD)^g^	0.5 (1.0)	0.4 (0.8)	0.4 (0.6)	0.4 (0.5)	0.1 (−0.2, 0.3)	0.64
24-h recalls,^e^ *n* (%)^f^	64 (96)	66 (97)	65 (97)	58 (85)		
All vegetables, M (SD)^g^	4.9 (3.1)	4.4 (2.9)	4.4 (2.0)	5.2 (3.9)	1.0 (0.2, 1.9)	**0.02**
Dark green	1.4 (1.6)	1.3 (1.6)	1.3 (1.1)	1.5 (1.5)	0.2 (−0.2, 0.7)	0.33
Deep yellow	0.3 (0.4)	0.4 (0.6)	0.3 (0.4)	0.5 (0.8)	0.2 (0.0, 0.4)	**0.03**
Tomato	0.9 (1.0)	0.6 (0.7)	0.7 (0.7)	0.7 (0.9)	−0.0 (−0.3, 0.2)	0.77
Other	2.4 (1.9)	2.0 (1.9)	2.1 (1.6)	2.5 (2.3)	0.5 (−0.1, 1.1)	0.08
Beans (not targeted), M (SD)^g^	0.3 (0.5)	0.3 (0.6)	0.3 (0.5)	0.2 (0.4)	−0.0 (−0.2, 0.1)	0.60

Boldface indicates statistical significance

^a^Sample sizes (*n*) for baseline and post-randomization values reported represent complete data

^b^Main effect of app from linear mixed model, using an intention-to-treat analysis with multiple imputation for missing data

^c^Measured at baseline and 8 weeks post-randomization

^d^Primary outcome defined *a priori*

^e^Measured at baseline and 5 weeks post-randomization

^f^Completed at least one recall

^g^Servings per day, unless otherwise noted

**Fig. 2:**
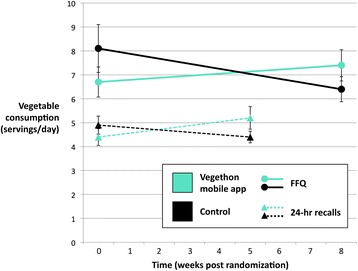
Changes in daily vegetable consumption. Mean ± SE, *n* = 135. Measured by FFQ (baseline to 8 weeks, *solid lines*) and 24-h recalls (baseline to 5 weeks, *dashed lines*)

Primary and secondary outcome measures of daily vegetable consumption at baseline and post-randomization and adjusted group differences are reported in Table 1. Changes in daily consumption of all vegetables are presented in Fig. 2. Daily consumption of all vegetables was significantly greater in the intervention versus control condition for both measures (adjusted mean difference: 2.0 servings; 95% CI: 0.1, 3.8, *p* = 0.04, Cohen’s d = 0.18 for FFQ; and 1.0 servings; 95% CI: 0.2, 1.9; *p* = 0.02, Cohen’s d = 0.20 for 24-h recalls). There was significantly greater consumption of cruciferous vegetables for FFQ (0.5 servings; 95% CI: 0.1, 0.8; *p* = 0.01) and of deep yellow vegetables for 24-h recalls (0.2 servings; 95% CI: 0.0, 0.4; *p* = 0.03). There were trends towards greater consumption of other vegetables for both measures (0.8 servings; 95% CI: -0.1, 1.7; *p* = 0.06 for FFQ; and 0.5 servings; 95% CI: -0.1, 1.1; *p* = 0.08 for 24-h recalls). Consumption of beans, a vegetable not targeted by the intervention, was not significantly different between the conditions (0.1 servings; 95% CI: -0.2, 0.3; *p* = 0.64 for FFQ; and −0.0 servings; 95% CI: -0.2, 0.1; *p* = 0.60 for 24-h recalls).

Baseline vegetable consumption was found to be a significant moderator of intervention effects (*p* = 0.002), in which the effect of the app on vegetable consumption increased as baseline vegetable consumption increased. It has been widely acknowledged in the statistical literature that ignoring a strong interaction can potentially lead to biased estimates of the main effect of treatment and to reduced power [32]. Indeed, when the model was refit including the app by baseline vegetable consumption interaction coefficient, we found substantially greater effect sizes for all vegetables (adjusted mean difference between treatment and control: 3.1 servings; 95% CI: 1.6, 4.6; *p* < 0.0005), including green vegetables (0.9 servings; 95% CI: 0.3, 1.4; *p* = 0.004) cruciferous vegetables (0.7 servings; 95% CI: 0.4, 1.0; *p* < 0.0005), dark yellow vegetables (0.3 servings; 95% CI: 0.1, 0.6; *p* = 0.01), tomatoes (0.3 servings; 95% CI: 0.1, 0.4; *p* = 0.001), and other vegetables (1.1 servings; 95% CI: 0.4, 1.9; *p* = 0.002).

**Fig. 3:**
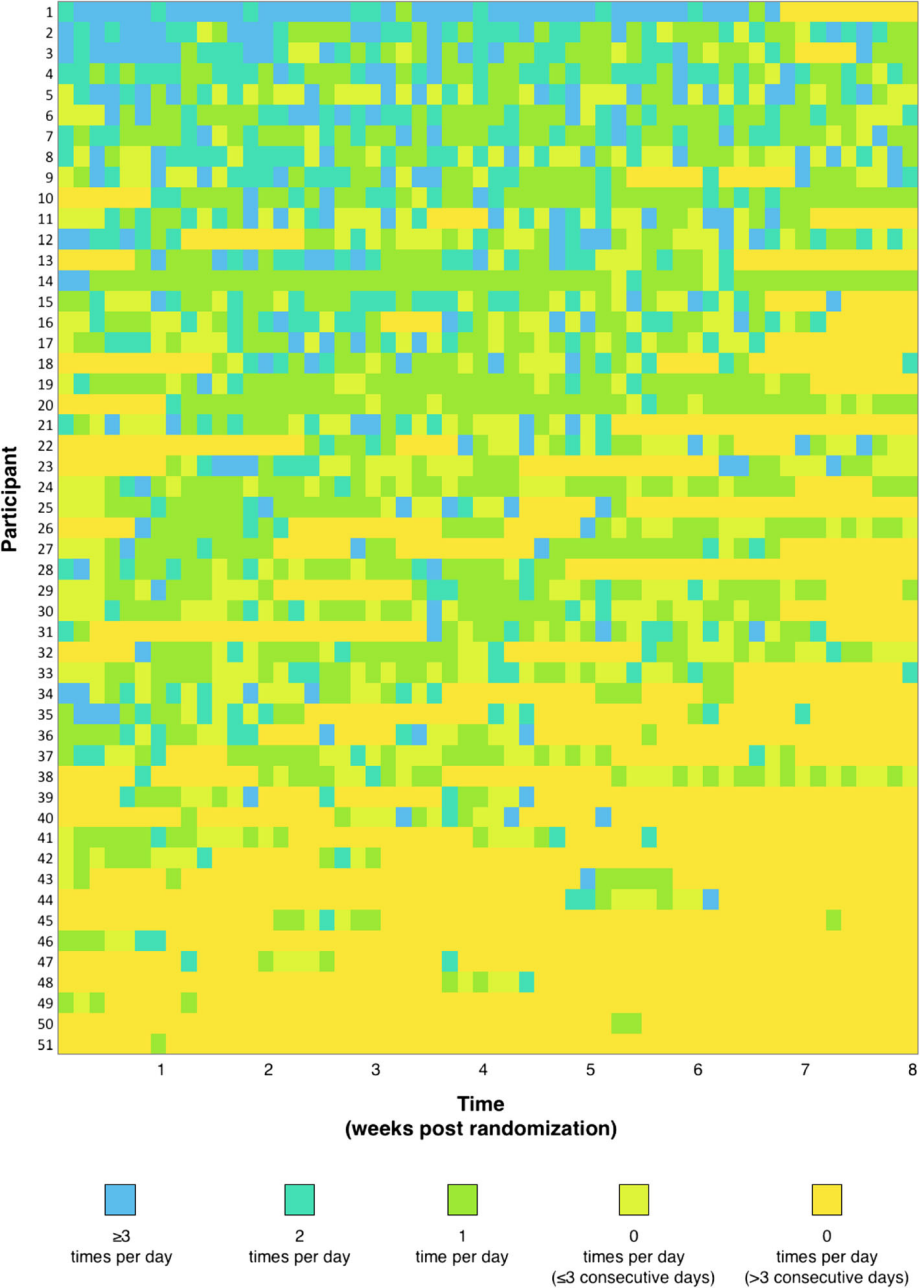
Frequency of vegetable logging among intervention condition. *n* = 51^1^. Participants sorted in decreasing order of logging frequency. ^1^Figure excludes 17 participants who did not log

Among the 51 (75%) intervention participants who used the app to log, participants logged their vegetable consumption 0.8 ± 1.1 times per day (mean ± SD). Daily frequency of vegetable logging over the course of the 8-week intervention period is presented in Fig. 3. There was a downward trend in frequency of logging behavior over time, from 0.9 ± 1.3 times per day during week one to 0.4 ± 0.8 times per day during week eight (mean ± SD). There was wide variation in logging frequency between individuals, ranging from 1.4 ± 1.2 to 0.2 ± 0.6 times per day in the upper and lower tertiles, respectively (mean ± SD). App usage was weakly correlated with changes in vegetable consumption (Spearman’s rank correlation coefficient: 0.05). However, among the subgroup of intervention participants above the 50th percentile of vegetable consumption at baseline (i.e., those who benefited most from the app), there was a larger correlation between app usage and changes in vegetable consumption (Spearman’s rank correlation coefficient: 0.2).

Participants who used the app at least once (*n* = 51) reported positive experiences with the intervention, including strongest agreement with the statements: “Vegethon has made me aware of how few vegetables I eat”; “I have found Vegethon easy to use”; and “Vegethon has helped me track my vegetable consumption” (4.1 ± 1.0, 3.8 ± 1.1, and 3.7 ± 1.1 [mean ± SD], respectively, on a 1–5 scale, with 5 = strongly agree and 1 = strongly disagree). They reported strong disagreement with the statements: “There has been too little training on how to use Vegethon”; “Overall, Vegethon was distracting”; and “Overall, using Vegethon required too much of my time” (2.1 ± 1.1, 2.3 ± 1.2, and 2.3 ± 1.1 [mean ± SD], respectively).

## Discussion

The theory-based Vegethon mobile app intervention led to significantly greater daily consumption of vegetables among overweight adults attempting weight loss maintenance. Participants randomized to receive the Vegethon app reported consuming an average of two more servings per day than controls, measured with the FFQ after 8 weeks. Participants randomized to receive the Vegethon app also reported consuming an average of one more serving per day than controls when measured with 24-h dietary recalls after 5 weeks, a secondary measure of vegetable consumption. The consistency of these results using two different measures of daily vegetable consumption assessed at two time points during the study lends further credibility to the study’s findings. Differences were not observed between groups in reported consumption of beans, a vegetable type not targeted by the intervention. This finding suggests the observed effects are unlikely to be explained by a differential social desirability bias leading to an over-reporting of all vegetables by participants who received the intervention. This stand-alone mobile app was evaluated in the context of an intensive weight loss trial in which consumption of high quality whole foods and vegetables was emphasized. The efficacy of the app beyond the effect of the parent weight loss intervention may suggest a potentially high degree of potency of the mobile app intervention or a synergistic effect between the two. These effects may be due to the advantages inherent to mobile-based interventions, including timely feedback, personalization, and daily interaction [9], as well as the theory-based nature of the intervention. This is the first study to demonstrate in a rigorous randomized controlled trial the efficacy of a stand-alone mobile app in increasing vegetable consumption.

Baseline vegetable consumption was a significant moderator of intervention effects, in which the effect of the mobile app on vegetable consumption was greatest among those who reported greater consumption of vegetables at baseline. This finding can inform future studies of Vegethon, and possible other interventions to increase vegetable intake, to test this observation directly by stratifying randomization on baseline vegetable consumption or by designing and testing different interventions for different groups defined by their prior vegetable consumption. Examination of the patterns of change over the course of the study suggests that, overall, the mobile app increased and maintained higher vegetable consumption in the intervention condition, compared to a reduction in vegetable consumption in the control condition (Fig. 2). This pattern may be explained because participants received the mobile app 5–7 months into a weight control program in which they had already made changes to their diets, including their vegetable intakes. It also highlights the importance of using a randomized controlled design to evaluate the efficacy of behavior change interventions, as an observational study of the mobile app may have been misleading, missing the large effect. After finding that baseline vegetable consumption moderated the impact of the intervention, an exploratory analysis including the baseline vegetable consumption by app interaction in the model showed even larger effects sizes, with significant increases in consumption of all vegetables, including green vegetables, cruciferous vegetables, dark yellow vegetables, tomatoes, and other vegetables. Future studies can be designed to account for the effect moderation of baseline vegetable intake. Given the improved health outcomes associated with greater vegetable consumption, we believe the observed effect sizes would be of clinical significance.

The intervention achieved reasonably high rates of engagement and was found to be easy and enjoyable to use. App usage showed a weak dose-response relationship with overall treatment effect but was more strongly associated with changes in vegetable consumption among the subgroup of participants who reported consuming more vegetables at baseline. This finding suggests that the effect of the mobile app is tied not only to frequency of use but also to individual participant characteristics.

The results of this study indicating the efficacy of the Vegethon intervention confirm those of the earlier Vegethon pilot study [18], in which increases in daily vegetable consumption among overweight adults were similarly observed. Effect sizes were substantial but smaller than those observed in the initial pilot study, as is typical when larger, more definitive trials follow small pilot studies [33]. However, they compare favorably with the only other known randomized study of a mobile app targeting vegetable consumption [15]. The theory-driven nature of the app, including goal-setting and self-monitoring behavior widely acknowledged to be critical components of behavioral mHealth interventions [11], may have led to greater behavioral changes than observed previously. These increases in vegetable consumption were observed among participants who already had a relatively high baseline vegetable consumption compared to the U.S. national average [5], due in part to their concurrent participation in a weight loss trial emphasizing healthier eating including vegetable consumption. Further studies are indicated to examine the effect of Vegethon and similar technologies among populations with lower vegetable consumption who are not concurrently enrolled in a weight loss trial. Overall, these findings align with those found in other investigations of mobile apps to change health behaviors, in which acceptability and initial efficacy has been similarly demonstrated [14].

These results warrant further research, as increases in vegetable consumption may lead to changes in overall diet composition and weight loss, even in the absence of specific guidance to decrease consumption of other foods [34]. For example, in a study among overweight adults by Norman et al., [35] a short message service (SMS) intervention that increased vegetable consumption led to weight loss. Such interventions that focus on the inherent benefits of the target behavior itself (e.g., increasing vegetable consumption) may lead to more sustained behavior changes than those focusing on longer-term goals (e.g., weight loss) [20]. Given the high and growing prevalence of overweight in the United States, and the fact that people in higher weight categories are more likely to develop chronic diseases associated with excess weight, strategies to reduce modifiable risk factors including diet among overweight adults are needed [36]. This study demonstrating the efficacy of a mobile app to increase vegetable consumption among motivated overweight adults presents one such possible strategy that warrants further investigation. Further trials of theory-driven mobile technologies to more effectively produce health behavior changes are called for.

Among the strengths of this research were the theory-based development of the app, the rigorous methods and randomized controlled study design, the use of state-of-the-art FFQ and 24-h dietary recall outcome measures, high retention rates for the primary outcome measure, and substantial sample size in comparison to most mHealth studies. This study used multiple methods of assessing vegetable consumption, which has been recommended to inform a more complete understanding of true intake [24]. Participants enrolled in the evaluation of this mobile intervention were concurrently enrolled in a weight loss trial. Generalizability of these findings to other samples that are not enrolled in a weight loss trial and/or motivated to use a mobile app to increase their vegetable consumption is unknown. Although comparable to many nutrition intervention studies, the 8-week study duration provides an assessment only of relatively short-term intervention effects. A longer trial is warranted to determine whether the observed effects increase, decrease, or are sustained over time, and whether there are effects on weight and other associated health benefits. As with all dietary assessment methods, there are inherent limitations to the adapted Harvard Food Frequency Questionnaire and 24-h recall measures including reliance on self-report. However, our finding that the differences in vegetable consumption between groups did not generalize to beans, a vegetable group not targeted by the intervention, lends support to the validity of the findings.

## Conclusions

These data show that a theory-based mobile app increased vegetable consumption among overweight adults attempting weight loss maintenance. Policy makers should consider the development and implementation of theory-based mobile apps as a low-cost, scalable, and effective intervention to support dietary behavior changes among overweight adults.

## Abbreviations

App: Mobile application
BMI: Body mass index
FFQ: Food Frequency Questionnaire
mHealth: mobile health
SMS: Short message service

## References

[1] Oyebode O, Gordon-Dseagu V, Walker A, Mindell JS (2014). Fruit and vegetable consumption and all-cause, cancer and CVD mortality: analysis of health survey for England data. J Epidemiol Community Health..

[2] Dauchet L, Amouyel P, Dallongeville J (2005). Fruit and vegetable consumption and risk of stroke: a meta-analysis of cohort studies. Neurology.

[3] Wang X, Ouyang Y, Liu J (2014). Fruit and vegetable consumption and mortality from all causes, cardiovascular disease, and cancer: systematic review and dose-response meta-analysis of prospective cohort studies. BMJ (Clinical research ed).

[4] U.S. Department of Agriculture and U.S. Department of Health and Human Services. Dietary guidelines for Americans, 2010. http://health.gov/dietaryguidelines/dga2010/DietaryGuidelines2010.pdf (accessed September 19 2015).

[5] Casagrande SS, Wang Y, Anderson C, Gary TL (2007). Have Americans increased their fruit and vegetable intake? The trends between 1988 and 2002. Am J Prev Med.

[6] Thomson CA, Ravia J (2011). A systematic review of behavioral interventions to promote intake of fruit and vegetables. J Am Diet Assoc.

[7] Cobiac LJ, Vos T, Veerman JL (2010). Cost-effectiveness of interventions to promote fruit and vegetable consumption. PLoS One.

[8] Pomerleau J, Lock K, Knai C, McKee M (2005). Interventions designed to increase adult fruit and vegetable intake can be effective: a systematic review of the literature. J Nutr.

[9] Klasnja P, Pratt W (2012). Healthcare in the pocket: mapping the space of mobile-phone health interventions. J Biomed Inform.

[10] Stephens J, Allen JK, Dennison Himmelfarb CR (2011). “smart” coaching to promote physical activity, diet change, and cardiovascular health. The Journal of cardiovascular nursing.

[11] Azar KMJ, Lesser LI, Laing BY (2013). Mobile applications for weight management: theory-based content analysis. Am J Prev Med.

[12] Riley WT, Rivera DE, Atienza AA, Nilsen W, Allison SM, Mermelstein R (2011). Health behavior models in the age of mobile interventions: are our theories up to the task?. Translational behavioral medicine.

[13] Pagoto S, Schneider K, Jojic M, DeBiasse M, Mann D (2013). Evidence-based strategies in weight-loss mobile apps. Am J Prev Med.

[14] Payne HE, Lister C, West JH, Bernhardt JM (2015). Behavioral functionality of mobile apps in health interventions: a systematic review of the literature. JMIR mHealth and uHealth.

[15] Nollen NL, Mayo MS, Carlson SE, Rapoff MA, Goggin KJ, Ellerbeck EF (2014). Mobile technology for obesity prevention: a randomized pilot study in racial- and ethnic-minority girls. Am J Prev Med.

[16] Breton E, Fuemmeler B, Abroms L (2011). Weight loss—there is an app for that! But does it adhere to evidence-informed practices?. Translational behavioral medicine.

[17] Helander E, Kaipainen K, Korhonen I, Wansink B (2014). Factors related to sustained use of a free mobile app for dietary self-monitoring with photography and peer feedback: retrospective cohort study. J Med Internet Res.

[18] Mummah S, Mathur M, King AC, Gardner CD, Sutton S. Mobile technology for vegetable consumption: a randomized controlled pilot study in overweight adults. JMIR Mhealth Uhealth. (in press) 10.2196/mhealth.5146 PMC4889871 27193036

[19] Mummah SA, King AC, Gardner CD, Sutton S (2016). Iterative development of Vegethon: a theory-based mobile app intervention to increase vegetable consumption. The international journal of behavioral nutrition and physical activity.

[20] Robinson TN, Dubé L, Bechara A, Dagher A (2010). Stealth interventions for obesity prevention and control: motvating behavior change. Obesity Prevention: The Role of Brain and Society on Individual Behavior.

[21] Robinson TN (2010). Save the world, prevent obesity: piggybacking on existing social and ideological movements. Obesity.

[22] Bandura A (1986). Social foundations of thought and action.

[23] Willet WC (1990). Nutritional epidemiology.

[24] Resnicow K, Odom E, Wang T (2000). Validation of three food frequency questionnaires and 24-hour recalls with serum carotenoid levels in a sample of African-American adults. Am J Epidemiol.

[25] Harvard School of Public Health. Nurses Health Study II Questionnaire. 2003. http://www.channing.harvard.edu/nhs/questionnaires/pdfs/NHSII/2003.PDF (accessed September 19 2015).

[26] Park SK, Tucker KL, O'Neill MS (2009). Fruit, vegetable, and fish consumption and heart rate variability: the veterans administration normative aging study. Am J Clin Nutr.

[27] Liu S, Manson JE, Lee IM (2000). Fruit and vegetable intake and risk of cardiovascular disease: the Women's health study. Am J Clin Nutr.

[28] Feskanich D, Sielaff BH, Chong K, Buzzard IM (1989). Computerized collection and analysis of dietary intake information. Comput Methods Prog Biomed.

[29] Johnson RK, Driscoll P, Goran MI (1996). Comparison of multiple-pass 24-hour recall estimates of energy intake with total energy expenditure determined by the doubly labeled water method in young children. J Am Diet Assoc.

[30] King AC, Hekler EB, Grieco LA (2013). Harnessing different motivational frames via mobile phones to promote daily physical activity and reduce sedentary behavior in aging adults. PLoS One.

[31] Cohen J (1992). A power primer. Psychol Bull.

[32] Kraemer HC, Wilson GT, Fairburn CG, Agras WS (2002). Mediators and moderators of treatment effects in randomized clinical trials. Arch Gen Psychiatry.

[33] Ioannidis JP (2005). Contradicted and initially stronger effects in highly cited clinical research. JAMA.

[34] Mytton OT, Nnoaham K, Eyles H, Scarborough P, Ni MC (2014). Systematic review and meta-analysis of the effect of increased vegetable and fruit consumption on body weight and energy intake. BMC Public Health.

[35] Norman GJ, Kolodziejczyk JK, Adams MA, Patrick K, Marshall SJ (2013). Fruit and vegetable intake and eating behaviors mediate the effect of a randomized text-message based weight loss program. Prev Med.

[36] Must A, Spadano J, Coakley EH, Field AE, Colditz G, Dietz WH (1999). The disease burden associated with overweight and obesity. JAMA.

